# Psychobiotics in Aquaculture: Harnessing the Microbiome–Gut–Brain Axis for Stress Management and Production Enhancement in Fish

**DOI:** 10.3390/ani15182726

**Published:** 2025-09-18

**Authors:** Mikhail Nikolaevich Churilov, Evgeniya Valeryevna Prazdnova, Dmitry Vladimirovich Rudoy

**Affiliations:** Research Laboratory «Agrobiotechnology Center», Don State Technical University, Gagarina Sq. 1, Rostov-on-Don 344002, Russia; eprazdnova@donstu.ru (E.V.P.); rudoy.d@gs.donstu.ru (D.V.R.)

**Keywords:** psychobiotics, aquaculture, microbiome–gut–brain axis, stress management, fish welfare, probiotics, sustainable aquaculture

## Abstract

Intensified aquaculture production has significantly increased farmed fish exposure to various stressors that compromise welfare and productivity, demanding innovative approaches to enhance sustainable production. Aquaculture environments subject fish to multiple stressors. These include high-density housing, handling, transportation, and fluctuating water quality. Such stressors activate the hypothalamic–pituitary–interrenal axis, resulting in a cascade of endocrine responses. This subsequently impairs feeding behavior, growth performance, and immune function. Psychobiotics exert beneficial effects by producing neuroactive compounds, including gamma-aminobutyric acid, short-chain fatty acids, serotonin, and dopamine. Key bacterial genera such as *Lactobacillus*, *Bifidobacterium*, *Bacillus*, and *Enterococcus* demonstrate significant psychobiotic properties. Experimental evidence from zebrafish and commercially relevant species demonstrates that psychobiotic interventions consistently reduce cortisol levels, normalize stress responses, and improve behavioral outcomes. These effects occur through the microbiome–gut–brain axis, a bidirectional system connecting the nervous and gastrointestinal systems by neural, endocrine, and immune pathways. Nevertheless, significant challenges persist, such as species-specific efficacy, limitations in mechanistic understanding, and the need for standardized evaluation protocols. This review examines psychobiotics as promising interventions for stress management in aquaculture systems. We conclude that psychobiotics may offer a sustainable approach for mitigating aquaculture stress responses and enhancing both fish welfare and production efficiency through targeted microbiome modulation.

## Simple Summary

Aquaculture production efficiency is limited by inherent industrial stressors. Psychobiotics are a novel group of probiotics that possess psychoactive properties and the ability to mitigate stress reactions. At present, the variety of discovered psychobiotics is limited and may not be well-adapted to the intestinal microbiota of farmed fish species. In this review, we summarize the mode of action of psychobiotics and the current state of psychobiotic research in teleost fish models, and we discuss directions for the identification and development of novel psychobiotics aimed at enhancing fish welfare and improving aquaculture performance.

## 1. Introduction

More than half of all fish and aquatic animal products now come from aquaculture, whose production scale surpassed that of wild capture fisheries in 2022. The production of capture fisheries has remained relatively constant since the late 1980s, fluctuating between 86 and 94 million tons annually. In contrast, aquaculture has achieved remarkable growth. It has been growing by 3.7 to 6.1 percent annually since the 2000s, ultimately reaching 94 million tons in 2022. The growth of aquaculture directly parallels rising global population food demand, with per capita consumption rising from 14.4 to 20.7 kg per year from the 1990s to the early 2020s. These trends appear most pronounced in the aquaculture production of freshwater species such as tilapia and river catfish, while production of marine fish shows more moderate expansion [1]. This expanding production scale of aquaculture has reshaped global food systems, thus strengthening economic development and nutritional security [2].

However, aquaculture intensification creates substantial challenges. Fish stress exposure and subsequent behavioral and metabolic changes directly compromise industry-relevant aquaculture productivity indicators. Chronic stress and acute stressor exposure, including high stocking densities, frequent handling, water quality fluctuations, and transport procedures, lead to decreased feeding activity, stunted growth, and increased feed conversion ratio [3]. Exposure to industrial stressors disrupts homeostatic balance and reallocates energy resources away from growth and reproduction toward physiological adaptation mechanisms, negatively affecting aquaculture productivity [4].

Probiotics have proven highly effective for addressing numerous challenges in aquaculture. Probiotic preparations serve as essential components in aquaculture nutritional supplements [5]. These preparations have established prominence as functional feed additives that improve intestinal health, enhance nutrient digestibility, modulate immune responses, and reduce reliance on antimicrobial treatments [6,7]. Industry-induced stress in cultured fish species, therefore, represents a logical next target for probiotic-based interventions, since probiotics can effectively modulate host physiology and enhance stress resilience [8].

Psychobiotics have recently emerged as a specialized subclass of probiotics that exert beneficial effects on host psychological health. While research has been concentrated on medical applications [9], compelling evidence supports psychobiotic applications for normalizing psychological and neurological processes in farm animals [10].

Fish, like other vertebrates, possess a sophisticated multi-component bidirectional communication system between the central nervous system and gastrointestinal tract: the microbiome–gut–brain axis (MGBA). Studies of probiotic preparations show opportunities for microbial modulation of neural processes, therapy for neurological and psychological disorders, and investigation of CNS impacts on intestinal microbiota [11,12].

Despite extensive probiotic research in aquaculture, psychobiotic applications has received limited attention in fish farming contexts. While recent reviews have thoroughly examined general probiotic applications in aquaculture [13,14] and MGBA interactions in vertebrates [15,16,17], comprehensive analysis of psychobiotics as targeted stress management interventions for farmed fish remains underexplored. This knowledge gap becomes particularly significant considering that fish MGBA research has concentrated predominantly on zebrafish models, with limited systematic translation to commercial species. Additionally, although psychobiotic research has advanced in mammalian systems, the distinct microbiological characteristics of teleost fish clearly indicate that species-specific approaches require further investigation for effective aquaculture applications [18]. In this review, we aim to address these gaps and evaluate the potential of psychobiotic applications for aquaculture fish to mitigate industrial stressor effects and thereby improve aquaculture productivity and welfare. Unless otherwise specified, our discussion focuses on teleost fishes, as they constitute the most commercially significant group in aquaculture.

## 2. Systemic Stress and Psychological Stressors in Aquaculture

Fish reared in aquaculture environments routinely encounter diverse stressors inherent to husbandry practices. These include technological manipulation, transportation, crowded housing conditions with restricted mobility, water quality fluctuations, and altered dietary patterns. These stressors may manifest acutely or chronically, and the artificial conditions characteristic of aquaculture systems typically fall short of optimal fish physiology requirements. Stressor exposure directly reduces aquaculture productivity, environmental tolerance, and food digestion compared to potential values under optimal conditions [19].

Distinguishing between enhancement of stress adaptation and stress response suppression proves essential when evaluating psychobiotic interventions in aquaculture. Stress responses represent adaptive physiological and behavioral mechanisms enabling fish to cope with environmental challenges and maintain homeostasis—these responses are essential for survival and are not inherently detrimental. In contrast, stressors are environmental factors (crowding, handling, water quality fluctuations, transport) that challenge homeostasis and become problematic when they exceed the fish’s adaptive capacity or persist chronically. Stress-mitigating interventions do not aim to eliminate stress responses, which would be maladaptive. They instead aim to optimize the fish’s ability to mount appropriate responses to stressors and prevent the transition from adaptive responses to pathological states [20,21].

Stress responses in fish closely parallel those of other vertebrates; however, fish possess specific features that require consideration when designing experimental protocols involving psychobiotics. The stress response in fish operates primarily through two central neuroendocrine pathways, alongside additional regulatory mechanisms discussed in subsequent sections.

### 2.1. HPI Axis Activation Under Chronic Stress

The neuroendocrine stress response in fish follows pathways analogous to those found in terrestrial vertebrates. However, fish lack separately developed adrenal glands; instead, equivalent endocrine function is performed by the interrenal tissue located in the head kidney. Consequently, the hypothalamic–pituitary–adrenal (HPA) axis in fish becomes the hypothalamic–pituitary–interrenal (HPI) axis, and the sympathetic adrenal–medullary (SAM) axis becomes the hypothalamic–sympathetic–chromaffin cell (HSC) axis [22].

Chronic stress exposure from unpredictable or uncontrollable environmental factors activates the HPI axis, resulting in increased bloodstream glucocorticoid levels [23]. Glucocorticoids, primarily cortisol, bind to intracellular receptors in various tissues. These receptors then function as transcription regulators by binding to glucocorticoid response elements in DNA [24,25].

Glucocorticoids have remarkedly broad regulatory action. This action can be categorized into five major functional domains. These are elevation of blood glucose levels, modification of behavior, somatic growth suppression, reproductive inhibition, and immunomodulation. Notably, corticosteroid-induced elevation of glucose occurs via stimulation of gluconeogenesis—a process that produces glucose from non-carbohydrate substrates, primarily proteins [26,27]. Corticosteroids induce tissue-specific glucose uptake alterations, redistributing energy resources towards the tissues involved in the stress response. Regarding immune function, elevated corticosteroids impair leukocyte proliferation and differentiation, decrease antigen presentation, and increase infection susceptibility [28].

Since cortisol represents the primary HPI or HPA system product, plasma cortisol levels and cortisol metabolism system activity remain the key chronic stress biomarkers in vertebrates [29,30,31]. Cortisol, however, is just one key element in the complex system of endocrine regulators within the HPI axis.

Additionally, urotensin I, secreted in the central nervous system and caudal neurosecretory system of fish in response to stress, is also a regulator of cortisol and adrenocorticotropic hormone release, reinforcing HPI axis regulatory output [32].

HPI axis stress response mechanisms generally activate adaptation processes and stressor avoidance, redistributing metabolic resources toward “emergency” functions [33]. While such adaptations serve to enhance survival, they compromise industrial animal performance, including aquaculture productivity, where increased anabolic efficiency, growth, development, reproduction, and immunocompetence are essential.

Although cortisol levels serve as the primary stress indicator in many studies, recent advances in multi-biomarker approaches using proteomics and metabolomics provide more reliable stress assessment than single-biomarker reliance. Panels including oxidative stress markers, immune-related cytokines, and neurotransmitter metabolites deliver a comprehensive stress evaluation while accounting for individual cortisol response variability [31,34]. Altered stress biomarkers, specifically reduced cortisol levels, following stress-mitigating interventions should be interpreted within the broader context of improved stress resilience and enhanced coping mechanisms rather than merely as indicators of ‘reduced stress’ [20].

### 2.2. Sympatho-Chromaffin Pathway and Acute Stress

Acute stressors predominantly activate the sympatho-chromaffin system, triggering the release of catecholamines (adrenaline, noradrenaline). This system responds to high-threat stressors including direct physical impacts and injuries, predator chase scenarios, anticipated predation risk, impaired oxygen transport, and acid-base imbalances [22,35]. Sympathetic nervous system stimulation represents the primary chromaffin cell activation route. Catecholamines rapidly affect cardiovascular and respiratory systems. Their effects include increased heart rate, elevated gill ventilation, enhanced oxygen transport, and adrenergic red blood cell stimulation to optimize oxygen delivery. Metabolic effects include liver glucose mobilization, gluconeogenesis stimulation, and lipolysis stimulation to provide readily available energy [36]. Hypoxic conditions, particularly when dissolved oxygen levels drop below 50–60% saturation, strongly trigger catecholamine release through chemoreceptors located in the gills [37,38].

Although sympatho-chromaffin responses are typically transient, with recovery occurring as rapidly as response induction, acute stress significantly impairs fish growth and reproduction, disrupting feeding behavior and food conversion efficiency [3,39]. Elevated noradrenaline specifically correlates with increased vulnerability to opportunistic and pathogenic bacteria, including *Yersinia ruckeri* and *Aeromonas hydrophila* [40,41].

### 2.3. Additional Regulatory Systems

Beyond HPI and HSC axes, several neurobiological and immunological pathways participate in teleost stress responses. These systems often interact with one another and with intestinal microbiota via the microbiome–gut–brain axis.

The serotoninergic regulatory system is deeply involved in fish stress responses, maintaining close relationships with HSC and HPI regulatory axes. It functions as a rapid-response regulatory network modulating both acute and chronic stress responses. The serotoninergic system response rate exceeds even catecholamine release response rates [42]. Moreover, social hierarchy factors in fish can activate this system. Chronic increase in serotonin production in fish results in decreased behavioral activity, including feeding behavior suppression. Importantly, individual variation in serotonergic receptor expression contributes to behavioral diversity within fish populations, including dominance hierarchy establishment [43].

The dopaminergic system exhibits lower reactivity compared to the serotoninergic system but functions in close coordination with serotonergic signaling. Dopamine signaling demonstrates similar individual sensitivity differences as serotonin signaling, and its activation reduces reproductive activity [44].

Brain-derived neurotropic factor (BDNF) is a major neurotrophin—a signaling protein deeply involved in neuron formation and development processes. Stress modulates BDNF expression which correlates closely with dopaminergic signaling [45]. Downregulation of BDNF following chronic stress is correlated with impaired cognitive function, disrupted social behaviors, and heightened anxiety-like responses, demonstrating its critical neuroprotective role [46].

Chronic stress promotes neuroinflammatory processes through microglial activation and the infiltration of peripheral immune cells such as monocytes and neutrophils. Activated microglial cells produce proinflammatory cytokines such as IL-1β and CCL2; generate immune makers such as Iba1, CD11b, CD86, TLR4, CD14, and CD68; and increase free oxygen species generation. These processes ultimately contribute to neural damage through affected neuron phagocytosis [47,48]. Furthermore, elevated systemic proinflammatory cytokine levels, including TNF-α and IL-1β, impair growth and development, negatively impacting aquaculture output [49].

Bilateral neuroimmune communication exists between the central nervous system (CNS), enteric nervous system (ENS) and the gastrointestinal (GI) tract as a whole. This communication allows non-inflammatory processes in the CNS to induce gastrointestinal disorders, microbiome changes and dysbiosis. Conversely, intestinal dysbiosis, often resulting from dietary changes, antibiotics or pathogen exposure can influence neurobehavioral outcomes and neuroimmune dynamics through microbial metabolite and immune mediator production [50].

## 3. The Microbiome–Gut–Brain Axis and Its Specifics in Fish

The gut–brain axis (GBA) in vertebrates represents a complex system of two-way communication between the central nervous system and the intestine. Through multiple regulatory pathways, the GBA integrates intestinal function and state with emotional and cognitive processes [51]. The regulatory pathways of the GBA include neural, endocrine, immune, metabolic, and epigenomic mechanisms [52,53].

In recent years, the GBA concept has evolved into the microbiome–gut–brain axis (MGBA), emphasizing the role of the intestinal microbiota as an active participant in host neuroendocrine and behavioral regulation [54]. Multiple studies demonstrate that severe intestinal dysbiosis or depletion of the intestinal microbiota results in profound behavioral and neurophysiological changes in vertebrates. Subsequently, numerous studies have emerged demonstrating the role of microbiome activity, its balance and diversity in the gut–brain axis regulatory mechanisms [55,56,57]. Research shows that the presence of specific microbial species in the intestinal microbiome exert both beneficial and negative effects on cognitive function, in particular through the synthesis of neuroactive compounds such as gamma-aminobutyric acid (GABA) and short-chain fatty acids (SCFAs) [58].

The MGBA regulatory pathways include closely interconnected neural, endocrine, immune, and metabolic processes [59,60]. Epigenetic regulation of the gut–brain interaction is also noted [53].

**Figure 1:**
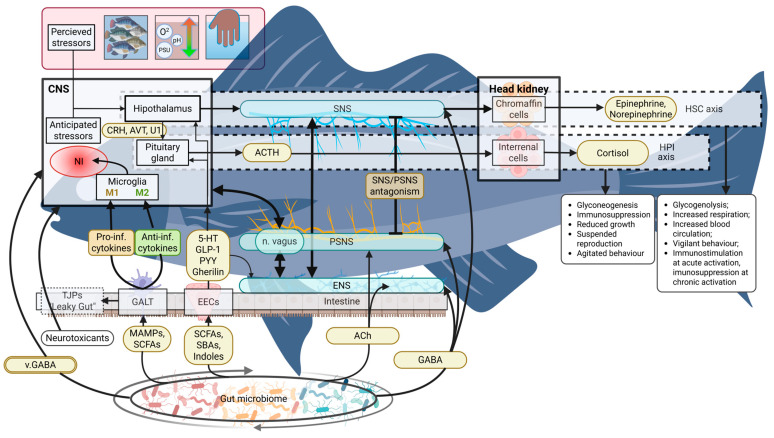
Interaction between microbiome–gut–brain axis and stress response pathways in teleost fish. Perceived or anticipated stressors activate the HSC and HPI neuroendocrine pathways triggering the release of associated hormones which facilitate a number of effects upon metabolism and behavior. Intestinal microbiota directly or indirectly affects both central neural regulation and the interplay between the divisions of the autonomic nervous system. Abbreviations: CNS, central nervous system; SNS, sympathetic nervous system; PSNS, parasympathetic nervous system; ENS, enteric nervous system; CRH, corticotropin-releasing hormone; AVT, arginine vasotocin; U1, urotensin I; ACTH, adrenocorticotropic hormone; TJPs, tight junction proteins; GALT, gut-associated lymphoid tissue; EECs, enteroendocrine cells; MAMPs, microbe-associated molecular patterns; SCFAs, short-chain fatty acids; SBAs, secondary bile acids; ACh, acetylcholine; GABA, gamma-aminobutyric acid; v.GABA, vesicular gamma-aminobutyric acid; NI, neuroinflammation; M1, pro-inflammatory microglial activation state; M2, anti-inflammatory (or neuroprotective) microglial activation state [61].

Figure 1 provides an overview of microbiome–gut–brain axis and stress response pathway interactions in teleost fish.

### 3.1. The Nervous System Component

The enteric nervous system (ENS), embedded within the intestinal wall, functions as an intrinsic division of the autonomic nervous system. Teleost ENS shows lower differentiation compared to mammalian ENS. It is distributed diffusely, without forming ganglia, and resides in the intermuscular intestinal layer. Teleost ENS contains intermuscular nerve plexus, but lacks both submucosal layer and submucosal nerve plexus. The enteric nervous system operates as a distinct element of the autonomic nervous system. It controls multiple gastrointestinal tract functions. These include peristalsis, secretion, blood flow, and immune responses [62,63].

The central neural communication component within the MGBA is the vagus nerve, which provides rapid regulatory signal transmission between the CNS and GI tract [64]. The vagus nerve facilitates integration of hormonal, mechanical, chemical, and microbial signals from the intestine via afferent fibers and modulates intestinal function through efferent outputs. It also closely interfaces with enteric neurons, enteroendocrine cells, and microbial metabolites [65,66].

### 3.2. Endocrine Signaling Within the MGBA

In hormonal regulation, the HPI axis in fish is analogous to the HPA axis of higher vertebrates. CNS exposure to various factors triggers corticotropin-releasing hormone (CRH) production by the hypothalamus, which stimulates adrenocorticotropic hormone (ACTH) production by the pituitary gland, which controls the production of corticoid hormones by the interrenal tissue. Corticoids, primarily cortisol, affect numerous body physiologic functions, including gastrointestinal tract functioning. Vasotocin, isotocin, urotensins, and melatonin serve as important hormonal regulators in fish, closely linked to the HPI axis operation [67]. The gastrointestinal microbiota modulates the secretion of cortisol, and provides necessary substrates for synthesizing hormones of this regulatory system [68].

Serotonin (5-hydroxytryptamine, 5-HT) is one of the major neurotransmitters involved in regulating behavior, homeostasis, and gastrointestinal functions, including peristalsis [69]. In mammals, the majority of peripheral serotonin is produced by enterochromaffin (EC) cells within the intestinal epithelium under nervous system regulation of chemical and mechanical factors. In teleosts, some ambiguity exists regarding the presence and differentiation degree of specialized enterochromaffin cells in different species, but the gastrointestinal tract remains a major serotonin source, with release subject to similar regulatory processes [70,71]. Importantly, microbial metabolites such as SCFAs stimulate the expression of tryptophan hydroxylase 1—the rate-limiting enzyme in serotonin biosynthesis—emphasizing the microbiota’s role in serotonergic regulation [69].

### 3.3. Immune Signaling and Intestinal Barrier Integrity

The gut-associated lymphoid tissue (GALT) of teleost fish comprises a diverse immune cell population, including T and B lymphocytes, eosinophils, macrophages, and dendritic cells, similar to other vertebrates [72,73]. This mucosal immune system maintains intestinal homeostasis and modulates systemic immune responses via cytokine signaling. Proinflammatory cytokines such as interleukin-1β (IL-1β), interleukin-6 (IL-6), and tumor necrosis factor-alpha (TNF-α) mediate microglial cell activation and neuroinflammatory processes within the CNS [74]. Additionally, intestinal immune function is essential to maintaining the barrier function of the intestine. Intestinal barrier integrity disruption—often termed “leaky gut”—increases permeability to microbial toxins and immune triggers. This promotes neuroinflammation, leading to altered behavior and stress physiology in fish [10].

### 3.4. Distinct Characteristics of the Fish Intestinal Microbiota

The phylogenetic composition of the fish microbiota exhibits distinct taxonomic features compared to mammalian microbiota. Notably, members of the phyla *Fusobacteriota*, *Firmicutes* (*Bacillota*) and *Proteobacteria* (*Pseudomonadota*) dominate the fish GI tract across numerous species and environmental types [75,76,77]. Intestinal microbial composition is shaped by a multitude of factors, including species, diet, geography, habitat type, and water chemistry [78,79,80]. Marine and freshwater fish exhibit different microbiome compositions, reflecting adaptation to different salinity levels and environmental conditions [79].

Marine herbivorous fish microbiota exhibits notable functional specialization, including producing numerous carbohydrate-active enzymes (CAZymes) and sulfatases for digesting complex sulfated polysaccharides from macroalgae [81]. These enzymes target sulfated polysaccharides that are absent or minimal in terrestrial environments [80]. Carnivorous species microbiota also demonstrates adaptations to associated substrates and produces proteases, chitinases, and lipases [82]. Fish microbiota shows high taxonomic variability yet maintains functional homogeneity—different sets of microbial species may inhabit individual fish from specific environments, but their core digestive and immunological processes remain stable across different microbial assemblages [83].

General overview of wild fish intestinal microbiota composition is provided in Table 1. It features particular genera identified as essential functional parts of fish intestinal microbiomes amongst hosts of various habitats and trophic categories, as proposed in comprehensive studies and overviews in this area.

Current accumulated evidence provides a foundation for characterizing typical intestinal microbiota composition in teleost fish. Furthermore, this knowledge facilitates hypothesis generation regarding potential impacts of probiotic and psychobiotic administration on teleost intestinal microbiome structural and functional qualities.

## 4. Psychobiotics: From Concept to Application

### 4.1. Definition and Mechanisms of Action

Psychobiotics are defined as live microorganisms that, when consumed in optimal amounts, benefit the psychological health of the host [93]. Initially conceptualized as therapeutic agents for psychiatric disorders, psychobiotics have been described as “living psychotropic drugs” capable of influencing central nervous system function [94]. Some researchers propose expanding the concept of psychobiotics to include prebiotics and other microbiome-modulating interventions that achieve beneficial neurological effects through GI and CNS interactions [95,96].

Psychobiotic activity mechanisms within the MGBA include several interconnected pathways: direct synthesis of neuroactive metabolites [97], immunomodulation [98], regulation of neuroendocrine responses [99], and enhancement of intestinal barrier integrity to prevent neurotoxic substance translocation [100].

*Lactobacillus* and *Bifidobacterium* bacteria, typical in probiotic preparations, have undergone the most extensive study for psychobiotic effects across a broad range of model organisms. Additionally, specific strains of *Lactococcus lactis*, *Bacteroides fragilis* [9,101] are sometimes qualified as psychobiotics. Furthermore, bacteria of the genera *Enterococcus*, *Streptococcus*, and *Escherichia* demonstrate documented capacity for neuroactive metabolite production [102,103,104,105,106]. *B. subtilis* has also been investigated as a psychobiotic alleviating neurodegenerative disease progression in model organisms [107,108].

Psychobiotic research in aquatic species requires important terminological distinction. Researchers explicitly using the term “psychobiotics” explore their effects in various, mostly mammal, animal models, whereas fish studies often employ the broader term “probiotics”, even when observing behavioral and MGBA-related effects that classify the investigated strains as psychobiotics.

### 4.2. Neuroactive Metabolites and Their Producers

Multiple bacterial strains influence the GBA in teleost fish through neuroactive metabolite production, representing a primary psychobiotic action mechanism.

Gamma-aminobutyric acid (GABA) serves as the principal inhibitory neurotransmitter in vertebrate central nervous systems. GABA’s functional importance extends beyond the brain to peripheral tissues, including the intestine, where it modulates gastrointestinal motility, secretions, and immune function through diverse receptor subtypes with distinct pharmacological properties [109]. Evidence suggests that changes in both circulating and brain levels of GABA are associated with alterations in intestinal microbiota composition, positioning GABA as a potent mediator of the GBA [110].

Importantly, while GABA itself cannot cross the blood–brain barrier (BBB), recent research suggests that peripherally produced GABA influences brain function indirectly. These influences are achieved through multiple mechanisms including vagal nerve signaling, enteric nervous system modulation [109,110]. Moreover, GABA itself was recently found capable to penetrate BBB through extracellular vesicle-mediated transport [111].

Short-chain fatty acids (SCFAs), primarily acetate, propionate, and butyrate, belong to the major anaerobic fermentation products of intestinal microbiota metabolism. These products represent the key mediators of the microbiome–gut–brain axis with profound neuroactive properties [112]. Several bacterial genera function as GABA producers with documented effects on behavior, stress responses, and neural function. These bacterial GABA producers include bacteria from the *Bacillus*, *Bacteroides*, *Bifidobacterium*, and *Lactobacillus* genera, with documented effects in finfish [76,82,87,89,110,113,114,115,116].

Unlike GABA, SCFAs easily cross the blood–brain barrier via monocarboxylate transporters (MCTs) expressed in cerebral endothelial cells, allowing direct brain tissue interaction and enabling modulation of microglial activity, neuroinflammation, and brain function. SCFA significance extends beyond their metabolic functions as energy substrates, demonstrating remarkable capacity to strengthen blood–brain barrier integrity, modulate neurotransmitter synthesis, influence neurotrophic factor levels, and regulate microglial maturation and function [115]. In teleost fish, representatives from the *Cetobacterium*, *Clostridium* and *Bacteroides* genera produce SCFAs within the intestine and exhibit regulatory properties towards MGBA [82,86,87,88,112,117,118].

The range of bacterial producers of metabolites capable to modulate the microbiome–gut–brain axis is presented in Table 2.

Certain important neuroactive metabolites were shown to be produced by psychobiotic bacteria in higher vertebrates, yet limited confirmation exists regarding reproducibility of these effects in finfish models. However, these effects can be expected given the commonalities in the endocrine functions and microbiome to ENS interactions.

Catecholamines are present in significant concentrations in the vertebrate gastrointestinal tract. They exist, primarily, in a biologically inactive conjugated form in germ-free animals; however, they convert to free form in animals with normal microflora, indicating microbial involvement in the catecholamine metabolism [120]. Notably, some pathogenic bacteria of the intestinal microflora use adrenergic signaling in quorum-sensing mechanisms, and, conversely, they are susceptible to host-derived catecholamine signals [121]. A number of neurotransmitter amines have been found in isolated cultures of intestinal microorganisms in vitro [122], and certain bacterial species possess enzymatic machinery analogous to that required for catecholamine synthesis [123] and catecholamine transporter homologs [124].

Serotonin is an important neurotransmitter necessary for the functioning of many psychological processes. The gastrointestinal tract serves as the primary peripheral source of serotonin, where it undergoes synthesis and storage in enterochromaffin cells and is released under neural and mechanical regulation. In microbiota transplantation experiments using germ-free animals, research shows that intestinal microorganisms stimulate the release of serotonin from enterochromaffin cells, and facilitate its conversion from conjugated to free form via microbial sulfatase and β-glucuronidase activities [125].

Histamine represents an important neurotransmitter and inflammatory mediator synthesized through microbial amino acid metabolism. While endogenous histamine production proves essential for normal physiological function, excessive microbial histamine synthesis can result in neurotoxic effects [126]. The intestinal microbiota is able to synthesize histamine [127] and other biogenic amines that provoke negative behavioral and psychological processes [128]. Biogenic amines of the microbiota directly affect numerous specific receptors of the intestinal epithelium, taking part in the regulation of intestinal function [129,130]. These receptors directly interact with the enteric nervous system and the GBA neural component [131].

## 5. Evidence Base for Psychobiotic Effects in Fish

### 5.1. Foundational Evidence from Model Organisms

A substantial proportion of psychobiotic research is conducted using standard laboratory models, among which *D. rerio* is the most widely used representative of teleost fish. The zebrafish model offers good methodological coverage, which enables diverse investigations and the assessment of markers across multiple biological levels following well-established protocols. Furthermore, as with other models employed in translational research, robust approaches have been developed for working with gnotobiotic test animals in zebrafish. Nonetheless, reliance on this single organism introduces a model bias that constrains the direct applicability of findings to commercially relevant aquaculture species.

Germ-free and gnotobiotic *D. rerio* models allow researchers both to demonstrate the overall importance of the microbiome and to isolate the role of individual microbial taxa. In this context, the study by Davis et al. [132] is particularly illustrative, as it highlighted the role of the microbiome in shaping stress responses. Comparing germ-free larvae to conventional zebrafish larvae, the authors demonstrated that zebrafish without microbiota exhibit fundamentally disrupted stress responses. zebrafish larvae exhibit fundamentally disrupted stress responses, failing to mount appropriate cortisol increases following osmotic shock-induced stress. This deficiency extends beyond hormonal responses to encompass broader physiological dysfunction, including absent expression of critical stress indicators. Ultimately, germ-free larvae exhibit abnormalities at behavioral level. Importantly, these deficits were shown to be reversible. When microbe-free larvae were introduced into a non-sterile environment at least 24 h prior to testing, their stress markers and behavior patterns normalized. Notably, substantial recovery of stress responses was observed even in monoassociated larvae, where the presence of a single microbial species, *L. plantarum*, was sufficient to restore normal functions. Although this bacterium is not a natural component of the fish microbiota, the authors point out the resilience of the investigated *L. plantarum* strain.

In addition to germ-free models, value is also found in approaches where the intestinal microbiome has been disrupted through antibiotic treatment. In particular, oxytetracycline-induced dysbiosis in adult zebrafish reduced brain dopamine and impaired social interaction; *L. rhamnosus* administration restored neurotransmitter levels and normalized behavior, reducing anxiety behavioral indicators [133].

The advantages of standard models lie not only in the feasibility of working with gnotobiotic animals but also in the availability of diverse genetically modified lines that mimic various psychological disorders. The use of both germ-free zebrafish and its transgenic lines modeling autism spectrum disorders demonstrates the ability of lactobacilli to mitigate behavioral and developmental impairments, even when these impairments were driven by genetic factors [134]. Supported by analyses of gene expression related to serotonin, dopamine, and tryptophan metabolism, this work also provides mechanistic insights into the pathways through which psychobiotics exert their effects.

Outside controlled-microbiome models, taxonomic characterization of the microbiota in psychobiotic research is essential, since dysbiosis can act both as a trigger of stress and as a consequence of it. In a stress-induced dysbiosis model, *L. plantarum* was shown to exert beneficial effects on both microbiota composition and behavioral indicators of stress [135]. Probiotic supplementation restored the abundance of key taxonomic groups within the intestinal bacterial community. Similarly to a study published in the same year [132], the authors also evaluated a set of gene expression markers to clarify the mechanisms underlying psychobiotic action. Their findings indicated that the effects are mediated through modulation of GABAergic and/or serotonergic pathways. However, microbiota alterations induced by probiotics present researchers with the challenge of distinguishing the direct effects of the administered strain from those mediated indirectly through microbiota modulation.

Integrative studies that include both behavioral indicators and gene expression analyses demonstrate that probiotic supplementation with lactobacillar probiotics exerts a significant positive impact on anxiety-related behaviors while also revealing changes in underlying neural processes. Dietary administration of *L. rhamnosus* IMC 501 was found to up-regulate brain transcripts involved in serotonin biosynthesis, transport, receptor signaling, and degradation. These transcriptional shifts were accompanied by increased expression of *bdnf* in brain tissue, indicating enhanced synthesis of the neurotrophin BDNF, which acts as a functional synergist of the serotonergic system. At the behavioral level, probiotic exposure altered shoaling patterns, while in the intestine, tryptophan synthesis and receptor genes were downregulated [116]. The authors of this study also reported significant microbiota-level effects: not only did the relative abundance of lactobacilli increase, but the proportion of streptococci rose even more substantially.

Despite the complexity inherent to the phenomena being studied, some investigations are limited to behavioral assays alone. Within such studies, the administration of a combined probiotic formulation containing *L. rhamnosus* CECT8361 and *Bifidobacterium longum* CECT7347 has been shown to elicit a pronounced anxiolytic effect in the novel tank test [136]. However, the authors were unable to derive substantive mechanistic insights from these results, confirming only the expected behavioral outcomes. It is noteworthy that, as in other analogous cases, the conclusions are framed in the context of potential human application. Nevertheless, the limited understanding of the underlying mechanisms indicates a need for further targeted experimental investigation.

The investigation of potential psychobiotics should not be restricted to heuristic selection of candidate strains. Valderama and colleagues [137] advocate for a systematic approach that commences with in silico candidate identification. Through analysis of genomic databases, the authors selected strains capable of producing relevant metabolites and subsequently proceeded to derive supernatants containing these products, conduct metabolomic assays, and perform functional testing in zebrafish. This sequential, multimodal strategy enabled them to obtain a product with clear effects on GABAergic signaling at the gene expression level and validate its efficacy in behavioral assays. The authors contextualize their multi-omics methodology within translational research, incorporating specific selection criteria and focusing specifically on postbiotic products. Additionally, they highlight the need to isolate novel probiotic strains, recognizing that the initial step of their work is limited by currently available bacterial genomes.

It is important to evaluate the effects of probiotics on stress responses within a broad context that includes not only stress markers but also the downstream consequences related to growth and development [138]. The inclusion of such markers provides a more comprehensive understanding of how probiotics influence multimodal regulatory systems even when the scope of a study does not address purely psychobiotic interventions,

Some behavioral markers, such as feeding behavior, are especially important when attempting to translate findings from model organisms to aquaculture species. However, when examined in isolation, changes in feeding behavior can sometimes give unexpected results. Although *L. rhamnosus* has previously been considered a promising probiotic and psychobiotic candidate, it was shown that its inclusion into the diet of zebrafish reduces feeding activity and leads to hypoglycemic effects [139]. According to the authors, these effects primarily arise from the strain’s ability to produce short-chain fatty acids (SCFAs) and the regulatory roles of these metabolites. Notably, consistent with findings from an earlier study [116], the authors also observed an increase in streptococci in the intestinal tract, identifying *S. thermophilus* as the dominant species. All these effects were observed along with changes in intestinal micromorphology. Behavioral alterations stem from complex underlying mechanisms, and it is important to account for the wide range of physiological effects induced by MGBA-modulating compounds such as SCFAs.

### 5.2. Evidence from Commercial Aquaculture Species

While zebrafish studies provide mechanistic insights, translating these findings to commercial aquaculture species represents the critical test of practical applicability. Evidence from commercially relevant species, though more limited, demonstrates psychobiotic effects across phylogenetically diverse teleost groups.

A notable study on translating research in probiotic stress modulation to marine aquaculture species was conducted by Aerts and colleagues [140]. This work established a detailed gnotobiotic model of European sea bass (*Dicentrarchus labrax*) larvae and evaluated more species-specific probiotic strains, including *Vibrio lentus* isolated from the gastrointestinal tract of the same fish species and *Bacillus* sp. LT3. By incorporating a broad panel of cortisol metabolites and precursors, the authors were able to assess the effects of probiotics on the overall functioning of the HPI axis and interpret these findings within the context of larval growth and development. This demonstrates that the adaptation of gnotobiotic models to aquaculture-relevant fish species is feasible and provides deeper mechanistic insights that extend beyond conventional model organisms.

Studies in aquaculture-relevant fish species often include specific endpoints for stress regulation alongside industrially relevant parameters. For example, in European sea bass, dietary supplementation with *L. delbrueckii* was shown to reduce cortisol levels [141,142]. These changes were observed alongside improvements in production traits, such as survival rates and the expression of genes involved in muscle growth. A similar cortisol-lowering effect has been reported in gilthead sea bream (*Sparus aurata*) following supplementation with *L*. *fructivorans* and *L. plantarum* [143]. In this case, the authors demonstrated that probiotics mitigated cortisol levels both under normal conditions and in responses to pH-induced stress. Similar design was used in the study by Varela et al. [144], where stress was induced in sea bream via high stocking density, a major industrial stressor in aquaculture. In this work, although to a lesser extent, reductions in cortisol were observed in under the influence of an autochthonous probiotics belonging to the genus *Shewanella*, which is not a typical for such studies. However, despite the broad evaluation of metabolic and growth outcomes associated with probiotic supplementation, the precise mechanisms underlying cortisol modulation often remain unidentified.

Freshwater aquaculture species also show positive responses to treatments with potential psychobiotic effects. From a behavioral perspective, commercial probiotic formulations based on *B. amyloliquefaciens* were reported to enhance feeding activity and to reduce behavioral indicators of anxiety and aggression in Nile tilapia (*Oreochromis niloticus*) [145]. In the same species, endocrine markers showed that dietary supplementation with *Aspergillus oryzae* slightly reduced cortisol levels, both at the baseline and after exposure to hypoxic stress. Probiotic-mediated stress mitigation was also evident in immune parameters and in the expression of heat shock-related genes [146]. Significant cortisol level changes have been documented in other freshwater species as well. For example, the introduction of probiotic bacilli into the diet of adult African catfish (*Clarias gariepinus*) resulted in a nearly threefold reduction in blood cortisol levels. The authors further observed correlation between cortisol levels and formation of erythrocyte micronuclei [147]. In addition, probiotic supplementation in olive flounder (*Paralichthys olivaceus*) resulted in not only cortisol reduction but also to transcriptional changes in several genes related to neurotransmission [148].

In anadromous fish, such as salmonids, evidence for psychobiotic effects on behavior is more limited and shows mixed results depending on the bacterial strains used. For example, dietary supplementation with *Enterococcus thailandicus* 04-394 and *Lactobacillus* brevis ISCAR-07433 increased, rather than decreased, the motility of overcrowded Arctic char (*Salvelinus alpinus*) [149]. The authors attribute this response, along with growth retardation in these groups, to possible inflammation triggered by the bacteria. Conversely, supplementation with *L. plantarum* showed no adverse behavioral impact [150]. This study assessed only general locomotor activity in isolation from other stress-related responses. Although restricted-space mobility trials can serve as a model of stress induction, interpretation of such data remains limited without supporting endocrine or genetic markers.

A year later, another study attempted to integrate behavioral and transcriptomic analyses in juvenile Chinook salmon (*Oncorhynchus tshawytscha*) [144]. In contrast to other reports, administration of a multispecies probiotic (composed of three *Bifidobacterium*, *Lactobacillus*, and *Lactococcus* strains) produced no noticeable behavioral changes. Nevertheless, a broad transcriptomic survey of neuroactivity-related factors allowed for deeper insights into the mechanisms by which psychobiotics may influence salmonids. Importantly, the genetic background of salmonids, which have undergone a whole-genome duplication event, requires separate consideration when studying gene expression in this group as the authors suggest. Although research on psychobiotics in salmonids is not as extensive as in freshwater or marine species, these findings emphasize the importance of strain-level selection and host-specific validation in aquaculture applications.

Evidence from commercially relevant species demonstrates successful psychobiotic effect translation from laboratory models to production environments, with cortisol reduction emerging as the most consistent outcome across the investigated species. The diversity of effective strains spans beyond traditional probiotic genera, including fish-associated bacteria such as *V. lentus*, *S. putrefaciens*, and *A. oryzae*. This diversity and occasional underperformance of the traditional strains, suggests that host-microbiome compatibility may be equally important as specific metabolic capabilities. However, the occurrence of strain-specific adverse effects, such as inflammatory responses observed with certain *Enterococcus strains* in Arctic char, underscores the critical need for species-specific validation protocols in commercial aquaculture applications.

### 5.3. Synthesis of Evidence and Research Gaps

Accumulated evidence demonstrates psychobiotic effects across phylogenetically diverse fish species, with consistent patterns of cortisol reduction, neurotransmitter modulation, and behavioral normalization. However, several critical gaps limit translation to commercial aquaculture applications. Most studies employ *Lactobacillus* and *Bifidobacterium* strains despite their relatively low prevalence in fish intestinal microbiomes, raising ecological compatibility and persistence questions. Indigenous genera such as *Bacillus*, *Cetobacterium*, and *Bacteroides* show promising preliminary results but remain understudied.

Mechanistic understanding remains limited to correlation-based observations in most studies, with few investigations tracking specific metabolic pathways or testing targeted interventions. The requirement for live bacterial cultures rather than metabolites alone suggests complex host-microbe interactions beyond simple substrate provision.

Finally, environmental factors including water temperature, pH, salinity, and seasonal variations significantly influence psychobiotic efficacy but undergo rare systematic evaluation. This represents a critical knowledge gap for field applications where environmental conditions cannot be precisely controlled. In addition, as many studies feature limited, single-level stress marker observations, it restricts their capacity to provide comprehensive mechanistic insight.

## 6. Current Challenges and Future Directions

### 6.1. Current Limitations and Challenges

Although psychobiotic use and development represents a promising direction, several factors that limit experiments and applications in this area.

First, psychobiotic agents’ high species- and strain-specificity requires effort to obtain psychoactive probiotic preparation that produces necessary effects without compromising other areas of probiotic action and optimally integrates with the rest of host microflora. Experimental data dispersion between different models, conditions and doses requires confidence in reproducibility of the expected effect in the target organism.

Additionally, the mechanisms of action of psychobiotics remain poorly understood with many experimental studies noting only correlations between probiotic administration and behavioral effects, interpreting the action mechanisms cautiously.

Current understanding of psychobiotic mechanisms is limited by incomplete knowledge of the full range of bioactive metabolites that mediate microbiome–gut–brain axis communication. Recent high-coverage metabolomics and untargeted approaches reveal hundreds of previously uncharacterized compounds in host-microbe interactions [151], many possessing potential neuroactive properties yet remaining functionally unexplored in fish models and systems. This unexplored area of metabolomics constitutes a major gap, since comparative studies show fish-associated bacteria have species-specific metabolic profiles that go far beyond the well-known compounds such as GABA, SCFAs, and neurotransmitter precursors covered in this review. Research of psychobiotic metabolite delivery mechanisms is still incomplete, especially in light of recent findings that bacterial extracellular vesicles may transport metabolites across the blood–brain barrier [152].

Probiotic efficacy in aquaculture depends significantly on environmental factors including water temperature, pH, dissolved oxygen levels, salinity, and dietary composition. These parameters affect both bacterial viability and host stress physiology, potentially modulating psychobiotic effectiveness. Seasonal variations and water quality fluctuations may also require adaptive dosing strategies to maintain consistent benefits throughout production cycles. Psychobiotic action exhibits the same intraspecific variability as the psychological processes it modulates, thus reproducibility of the desired effect even within one species requires harmonization with other influences and environmental factors.

Addressing these limitations proves critical for successful psychobiotic implementation in aquaculture. Species- and strain-specificity, compounded by limited mechanistic clarity and environmental variability, necessitates more targeted yet interdisciplinary research approaches. Prioritizing collaborations between geneticists, microbiologists, and aquaculture practitioners could streamline practical, reliable psychobiotic intervention development.

Well-planned psychobiotic research should carefully address ethical, environmental, and safety considerations within its scope. Evaluation of potential antibiotic resistance transfer, allergenicity testing, and environmental impact studies require consideration for both safety concerns and to exclude potentially dangerous interventions early. Regulatory approval should follow established probiotic development pathways and incorporate additional neurobehavioral efficacy endpoints and specialized safety considerations for psychoactive microbial metabolites. This proves particularly important for genetically engineered psychobiotic strain development producing metabolites that may fall under stringent regulatory standards.

### 6.2. Methodological Approaches for Psychobiotic Development

When selecting psychobiotics for aquaculture species, researchers can pursue several approaches. First, they may utilize strains that demonstrate psychobiotic effects in fish models outside commercial aquaculture species (e.g., *Danio rerio*). Alternatively, they can focus on microorganisms already available as probiotic preparations for specific commercial species and screen for those with additional psychobiotic properties, or conduct proven strain modification.

Researchers generally consider psychobiotics for their action in pathological and suboptimal psychological states. Contrary to the studies with translational scope, the development of products for aquaculture assumes that the physiology of the host is not significantly impaired. When evaluating the effects of potential psychobiotics, it is worth considering that changes in animal behavior under probiotic exposure alone do not suffice to classify a strain as a psychobiotic. Gastrointestinal tract disturbances and pathologies can themselves cause behavioral changes in animals, including fish [135]. If a probiotic alleviates these disturbances, it may indirectly affect behavior, but its primary action may lie elsewhere. Therefore, understanding the specific action mechanisms of psychobiotics is needed to distinguish strains that directly affect neural processes, those that normalize regulatory markers and behavior indirectly (e.g., through microbiome modulation), and strains whose action is too condition-dependent for reliable employment.

Psychobiotic research must carefully differentiate between interventions that optimize adaptive stress responses versus those that inappropriately suppress necessary physiological reactions. Effective psychobiotics should enhance fish capacity to mount appropriate, proportional responses to genuine stressors while facilitating efficient recovery to baseline states. This contrasts with interventions that might blunt essential stress responses, potentially compromising fish ability to respond appropriately to environmental challenges [20,21]. Evaluation protocols should therefore assess not only stress response magnitude but also the appropriateness of stress responses to stressor intensity, as well as response duration and recovery efficiency. Multi-level assessment incorporating primary, secondary, and tertiary indicators provides more complete pictures of whether psychobiotic interventions genuinely enhance stress resilience rather than create maladaptive suppression of necessary physiological processes [21,153]. For widespread aquaculture psychobiotic adoption, clear differentiation between direct neuromodulatory actions and indirect gut health benefits proves essential. Accurate functional classification will guide both research and application, ensuring interventions selected for commercial use provide targeted benefits beyond general health improvements.

In light of the aforementioned considerations, we propose that the initial selection of microbial strains with potential applications in aquaculture should prioritize their capacity to synthesize bioactive compounds with broad neuroregulatory and metabolic functions. Such compounds in the first place include GABA, SCFAs and neurotransmitter precursors such as tryptophan-derived metabolites. Metabolite production can be rapidly assessed using established cultural and biochemical screening protocols, biosensory tests, and enzymatic colorimetric assays [154,155,156].

To ensure species-specific compatibility, candidate psychobiotics should undergo phylogenetic evaluation to match bacterial strains with the core microbiome composition of target fish species. Whenever possible it may be a good practice to aim for autochthonous strains derived from the target species and their immediate environment. Competitive exclusion assays should be conducted before major in vitro experiments. At in vivo investigations the microbiome composition should be monitored with cultural and genetic methods to evaluate the ability of psychobiotic candidates to establish stable populations within existing intestinal microbial communities without disrupting beneficial indigenous microorganisms.

Nevertheless, methodological caution is required during in vitro screening. Standard culture conditions may not accurately replicate the intestinal environment, including host signals and microbe interactions that affect metabolite production. Extensions to the established methods should be considered [157]. These approaches should include careful investigation and reproduction of the luminal chemistry, paying attention to the differences between intestinal compartments. Ex vivo models may also be applied if the researchers face the limitations of purely cultural methods.

Following biochemical screening, strains can be further evaluated through in vivo trials employing appropriate teleost models, focusing on their modulatory effects on host behavior, neuroendocrine stress axes, and immune parameters. These functional assessments may include behavioral assays (e.g., novel tank diving, color preference tests), serological biomarkers, and molecular analyses such as real-time qPCR profiling of stress- and neuroimmune-related genes. Studies involving germ-free and gnotobiotic animals have limited methodological coverage outside traditional laboratory models, yet adapting them to other animal models is possible. These approaches, and to some extent experiments involving antibiotic interventions, may provide deeper insight into the mechanism of action of psychobiotics, allowing the researchers to isolate direct and indirect modes of probiotic-host interaction.

Psychobiotic action manifests at multiple levels from immediate psychoactive metabolite production through indirect modulation to behavioral effects and further to practical benefits. Therefore, standardized psychobiotic evaluation protocols should develop through collaborative research institution efforts to ensure consistent methodology across studies. This standardization should include unified behavioral testing protocols, standardized stress challenge procedures, and harmonized biomarker assessment methods. As long as experimental evidence for psychobiotic action outside standard laboratory models stays scattered between the different hosts and strains investigated, it is important to design experimental studies in complementation to previous observations in this area. Screening methods must balance scientific rigor with commercial practicality. Streamlined protocols—ideally combining high-throughput screening with key functional assays—will prove necessary to encourage industry adoption without excessively increasing operational complexity or costs

### 6.3. Research Gaps and Future Opportunities

Experimental studies exploring psychobiotics in finfish remain relatively limited, placing fish-specific psychobiotics into a marginal position in psychobiotic research. These limited experimental studies have primarily evaluated *Lactobacillus*, *Lactococcus*, *and Bifidobacterium* strains, despite these bacterial genera not being characteristic of fish intestine microbiomes; thus, their stability in such environment may be questioned.

To date, *Lactobacillus* genus members represent the most extensively characterized bacterial group with documented psychobiotic properties. However, their prevalence within the teleost intestinal microbiota is relatively low, and their contribution to wider metabolic functions appears limited compared to other dominant taxa. By contrast, genera such as *Bacteroides*, *Bacillus*, and *Cetobacterium* are indigenous to the fish intestinal tract and have demonstrated the capacity to synthesize a variety of bioactive metabolites relevant for MGBA modulation and host physiological regulation. Despite this, their specific psychobiotic potential remains largely understudied.

Therefore, there is significant potential for deeper exploration of psychobiotic effects from GABA-producing strains of *Bacillus* and *Bacteroides*, as well as SCFA-producing strains of *Cetobacterium* and *Bacteroides*. *Bacillus*-based probiotics are of particular interest due to their ability to produce a wide range of beneficial metabolites. These include modulators of cellular stress [158], promoters of fish immune status [159], broad enzymatic functions [160]. In addition, bacteria of Bacillus genus are recognized for good persistence within the intestinal microbiome of aquaculture species [161]. The efficiency of paraprobiotics and postbiotics derived from *Bacillus* species at improving growth performance and immune functions and their positive microbiome modulation in various fish species also supports their potential for further research [162].

Future research should emphasize indigenous strains better suited to the fish intestinal environment, as these are more likely to persist and function effectively in production systems. Such candidates may provide advantages in stability, efficacy, and regulatory compliance compared to traditional probiotics.

Importantly, given current gaps in our mechanistic understanding of psychobiotic actions in fish, it remains possible that strains without confirmed metabolic outputs may still exert psychoactive effects by yet-unknown mechanisms. Therefore, it is recommended to include not only metabolic and immunological parameters, but also behavioral, genetic and endocrine endpoints. A comprehensive approach should enable reverse-tracing the psychobiotic effect to specific metabolites or host–microbiota pathways using integrative analyses. This includes gene expression assays and other transcriptomic methods.

At present, mechanistic exploration of psychobiotic function is most effectively pursued via metabolite profiling—focusing on compounds with known neuroactive, endocrine, and paracrine signaling roles such as GABA, serotonin precursors, and short-chain fatty acids. Nonetheless, additional layers of interaction—particularly those involving neuroimmune modulation, behavioral effects linked to complex microbial community reshaping, and even epigenetic regulation warrant further investigation.

Employing multi-omics and systems biology approaches will allow identification of new neuroactive metabolites and clarify psychobiotic action pathways in fish. These tools can hasten discovery-to-application timelines and support more targeted strain selection.

Moreover, only some psychobiotic effects and corresponding microbial metabolites were observed in teleost models, even though a broad spectrum of gut-associated microbes was shown to exhibit them in other vertebrates. Thus, a more integrative, systematic approach may help to fill in the gaps and uncover novel psychobiotic candidates among fish-associated microbiota with benefits for aquaculture welfare and productivity.

Bridging the gap between laboratory research and commercial application requires consideration of cost, scalability, and regulatory standards. Demonstrating economic and welfare gains, alongside ease of use, will be pivotal for real-world uptake of psychobiotics in aquaculture practices.

## 7. Conclusions

This comprehensive examination of psychobiotics in aquaculture reveals a transformative paradigm shift toward sustainable and welfare-focused fish farming practices. This emerging field represents a convergence of microbiome science, neurobiology, and aquaculture technology that holds significant promise for addressing the industry’s most pressing challenges while advancing environmental stewardship.

Only some psychobiotic effects and corresponding microbial metabolites were observed in teleost models, even though a broad spectrum of gut-associated microbes was shown to exhibit them in other vertebrates. Thus, a more integrative, systematic approach may fill in the gaps and uncover novel psychobiotic candidates among fish-associated microbiota with benefits for aquaculture welfare and productivity.

In this review, we provide the first systematic categorization of indigenous psychobiotic candidates specifically adapted to fish intestinal environments. Usually, such reviews focus predominantly on traditional probiotic genera (*Lactobacillus*, *Bifidobacterium*) with limited relevance to fish microbiomes, while we identify some “non-canonical” probiotic genera such as *Bacillus*, *Cetobacterium*, and *Bacteroides* as promising psychobiotic candidates with ecological compatibility and metabolite production capabilities in teleost systems. This represents a paradigm shift from applying mammalian-derived psychobiotics to fish toward developing fish-specific psychobiotic solutions.

Moving beyond the zebrafish-centric focus that has dominated previous fish microbiome research, we suggest synthesis from European sea bass, gilthead sea bream, Nile tilapia, African catfish, and salmonid species that demonstrate species-specific psychobiotic efficacy patterns.

We also demonstrate that psychobiotic interventions can simultaneously address multiple aquaculture challenges—stress management, disease resistance, growth optimization, and welfare enhancement—through targeted modulation of the microbiome–gut–brain axis. This integrative approach represents a fundamental advancement from single-function probiotics toward comprehensive psychobiotic strategies that optimize both fish neurophysiology and production outcomes.

Over the coming years, aquaculture psychobiotic research will likely evolve from proof-of-concept studies to comprehensive, systems-level strategies for optimizing fish health, stress tolerance, and production efficiency. Sophisticated screening platforms and integrated biological methods will drive the identification and functional validation of novel indigenous psychobiotic candidates, especially those capable of colonizing aquaculture-specific environments. Advances in formulation technology and microbial engineering may further enable tailored, robust products for different fish species and life stages. With regulatory harmonization and growing consumer demand for welfare-centered and sustainable aquaculture, psychobiotics could represent a core component of next-generation functional feeds, supporting both profitability and ethical practice across a rapidly expanding global industry.

## Figures and Tables

**Table 1:** Major bacterial genera of intestinal microbiome of teleosts and their significance.

Phylum	Genus	Prevalence in Fish Intestine	Functionality	References
*Actinomycetota*	*Corynebacterium*	Present in both marine and freshwater species	Contributes to nutrient metabolism; Produces vitamins and antimicrobial compounds	[79,84]
	*Micrococcus*	Present in both marine and freshwater species	Supports digestive processes; Produces antimicrobial compounds	[79,85]
*Bacillota*	*Bacillus*	Abundant across fish species	Spore-forming probiotic; produces digestive enzymes; has antimicrobial activity	[78,79]
	*Clostridium*	Common across fish species	Cellulase production; vitamin synthesis; carbohydrate fermentation; amino acid metabolism	[79,86]
	*Lactobacillus*	Moderately common in marine and freshwater fish; enhanced by probiotics	Probiotic; lactic acid production; immune modulation; pathogen inhibition; enzyme production	[79,87]
*Bacteroidota*	*Bacteroides*	Found in marine and freshwater fish; variable abundance; feed-associated	Polysaccharide degradation; SCFA production; vitamin B12 synthesis; bile acid metabolism; cellulolytic activity	[79,82]
	*Flavobacterium*	Common in marine and freshwater fish	Amylase, protease, chitinase production	[79]
*Fusobacteriota*	*Cetobacterium*	Dominant in freshwater fish; major across various species	Vitamin B12 synthesis; SCFA production; supports nutrition and immune function	[82,88]
*Mycoplasmatota*	*Mycoplasma*	Dominant in salmonids; can be majority of intestinal microbiota in rainbow trout	Potential mutualist; amino acid metabolism;	[89,90]
*Pseudomonadota*	*Aeromonas*	Common in marine and freshwater fish; can dominate intestinal microbiome in some conditions	Protease production; opportunistic pathogen; competes with beneficial bacteria	[82,91]
	*Enterovibrio*	Frequent in marine fish intestine; dominant in some species like milkfish	Nutrient processing; marine adaptation	[82,92]
	*Photobacterium*	Highly prevalent in marine fish; co-dominant with *Vibrio*	Chitinase production; protein metabolism; some are luminescent symbionts	[82]
	*Pseudomonas*	Prevalent in marine fish; reported in several carnivorous species; associated with lipase production	Protease/chitinase/lipase production;	[76,77,91]
	*Vibrio*	Dominant in marine fish, frequently reported across multiple species; up to 50% of viable bacteria in some marine fish	Protease and chitinase production; some species are beneficial, some are opportunistic pathogens; assists chitin/protein digestion	[79,82,92]

**Table 2:** Bacterial producents of MGBA-affecting metabolites in fish.

Phylum	Genus	MGBA-Relevant Metabolites	Fish Species	References
*Actinomycetota*	*Bifidobacterium*	GABA	Zebrafish	[114]
*Bacillota*	*Bacillus*	GABA	Multiple fish species, turbot	[76,115]
	*Clostridium*	SCFAs, butyrate, vitamins	Grass carp, turbot, various teleost species	[86,118]
	*Lactobacillus*	GABA	Zebrafish, rainbow trout	[89,113,119]
*Bacteroidota*	*Bacteroides*	GABA, SCFAs, tryptophan	Multiple marine fish species, zebrafish, threespine stickleback	[82,86,112]
*Fusobacteriota*	*Cetobacterium*	Vitamin B12, acetate, butyrate	Zebrafish, common carp	[88]
*Mycoplasmatota*	*Mycoplasma*	Amino acids	Limited evidence for MGBA effects in salmonids	[89,90]

## Data Availability

Not applicable.
